# Novel *GJB2* mutation c.188delT compound with c.235delC causing non-syndromic hearing loss in a Chinese family: A case report

**DOI:** 10.1097/MD.0000000000039266

**Published:** 2024-08-16

**Authors:** Yilun Tao, Zhipeng Hu, Dong Han, Wenxia Song, Lihong Wang, Haiwei Wang, Xiaoze Li

**Affiliations:** a Medical Genetic Center, Changzhi Maternal and Child Health Care Hospital Changzhi, Shanxi, China.

**Keywords:** frameshift variant, *GJB2* gene, hearing impairment, non-syndromic hearing loss (NSHL)

## Abstract

**Rationale:**

Congenital sensorineural hearing loss is a significant global health issue, primarily driven by genetic factors, such as mutations in the *GJB2* gene. This report presents a Chinese girl with congenital deafness and a novel mutation of the *GJB2* gene.

**Patient Concerns:**

A newborn Chinese girl exhibited signs of congenital deafness.

**Diagnosis:**

Congenital deafness was confirmed through comprehensive newborn hearing screenings that included otologic, audiologic, and physical examinations. Genetic analysis revealed a compound heterozygous mutation involving c.188delT and c.235delC in the *GJB2* gene, indicating a genetic basis for her hearing loss.

**Interventions:**

The patient underwent cochlear implantation, which resulted in stable auditory outcomes.

**Outcomes:**

Despite follow-up difficulties, stable auditory outcomes were achieved post-cochlear implantation, highlighting the potential efficacy of this intervention in *GJB2*-related hearing loss.

**Lessons:**

This case study enriches our understanding of *GJB2* mutations and underscores the critical role of genetic testing in diagnosing congenital sensorineural hearing loss. It emphasizes the necessity for early intervention and sustained interdisciplinary care to enhance the quality of life for patients with genetic hearing impairment.

## 1. Introduction

Hearing loss, the most common congenital sensory impairment, affects approximately 1 in 500 neonates and 1 in 300 children by age 4, profoundly impairing language acquisition and quality of life.^[1,2]^ The genetic basis of hearing loss is complex, involving numerous loci and genes underlying hereditary sensory neural non-syndromic hearing loss (NSHL).^[3,4]^ The *GJB2* gene (MIM 121011) was first identified in 1997 as being associated with autosomal recessive deafness type 1A (DFNB1A, OMIM #220290).^[5]^ Globally, *GJB2* mutations are exceedingly prevalent, accounting for up to fifty percent of NSHL cases.^[6,7]^

To address the prevalence of hearing loss and its potential repercussions, China has embarked on collaborative initiatives involving hearing screening and genetic analysis. Critical mutations that occur in genes linked to deafness, such as *GJB2*, *SLC26A4*, *GJB3*, and 12s rRNA, hold considerable importance. This screening method can accurately diagnose a substantial proportion (33%) of patients, specifically those belonging to the Han, Hui, and Tibetan ethnic groups in China.^[8,9]^ However, traditional genetic screening methods for deafness may not entirely identify the underlying cause in every patient, with an estimated 10% to 50% of patients remaining undiagnosed.^[8,10,11]^ Therefore, additional evaluation is required to determine the fundamental factors contributing to hearing impairment in these individuals.

In this study, we present a case of congenital deafness identified in a newborn. We identified a novel truncating variant, c.188delT, in combination with c.235delC in the *GJB2* gene in this patient.

## 2. Case report

**Figure 1.**
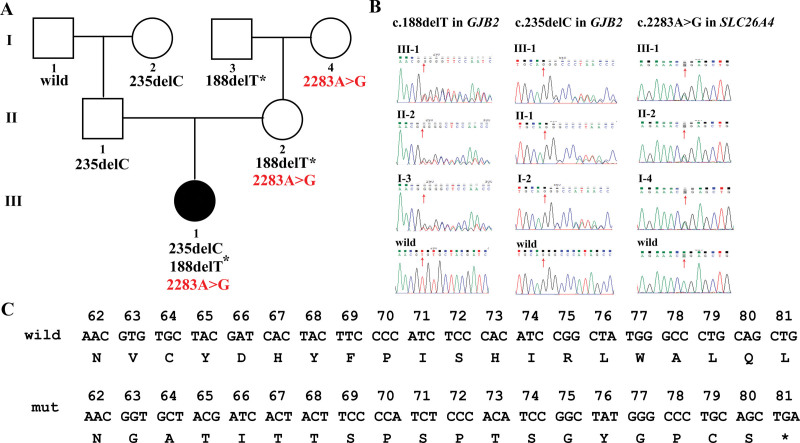
Genetic analysis of the *GJB2* and *SLC26A4* genes in a Chinese family with deafness. (A) Pedigree structure of the family with deafness. The round filled with black represents the deaf newborn. *GJB2* mutations are shown in black, while *SLC26A4* variants are shown in red. The novel mutation is marked with an asterisk. (B) Sequence analysis of the proband and her families. Red arrow indicates the location of variants Sanger sequencing result of c.235delC in *GJB2* gene were its complementary strand result, so the complementary base for c.235delC was G. (C) Amino acid sequences of wild-type and mutated proteins for the c.188delT variant in *GJB2* gene.

The girl was the first-born and only child of healthy, non-consanguineous Chinese parents (Fig. 1A). The birth length was recorded as 50 cm (75th percentile), and the birth weight was 2700 g (5th percentile) at 39 weeks of gestation. At birth, she failed the hearing screening in both ears. On day 6, assays for common deafness genes (4 genes and 9 pathogenic variants) were conducted using a hereditary deafness gene mutation detection kit and the microarray method (CapitalBio Technology Co., Ltd., Beijing, China). The testing outcomes revealed a solitary mutation in the *GJB2* gene (NM_004004.6), c.235delC (p.Leu79CysfsTer3), and an additional mutation in the *SLC26A4* gene (NM_000441.2), c.2283A > G (p.Thr761=). However, the patient had been diagnosed with bilateral sensorineural hearing loss at a previous external hospital.

To uncover the relevant genetic information, sequencing of the entire coding sequence of the *GJB2* and *SLC26A4* genes was conducted by Sanger sequencing. The peripheral blood samples from the proband, her parents, and other family members were collected. DNA samples of the proband and her family members were extracted from blood using the QIAamp DNA Mini Kit (Qiagen, China). PCR amplification of the coding (exon 2), noncoding (exon 1), and flanking intronic regions of the *GJB2* gene was conducted using the reported primers.^[12]^ The 30 μL PCR system including 15 μL 2X Phusion Master Mix (NEB, M0531S), 10 ng gDNA, 15 pmol forward and reverse PCR primers and 11 μL ddH2O. The PCR protocol was as follows: a denaturing cycle of 2 minutes at 98°C, 30 cycles of amplification (98°C for 30 seconds, 64°C for 30 seconds, 72°C for 30 seconds), and a final extension of 10 minutes at 72°C. For *SLC26A4*, the primers and procedures were described previously.^[13]^ Subsequently, the PCR products were transferred onto a 0.1% agarose gel, and the target DNA fragment was extracted. This fragment was prepared for Sanger sequencing on an ABI 3730 genetic analyzer (Applied Biosystems, Foster City Carlsbad), and Chromas 2.6.5 was used for identification.

A novel heterozygous deletion of 1 base pair (bp) in exon 2 (c.188delT) of the *GJB2* gene was detected in both the proband and her mother (Fig. 1B). The removal of this nucleotide sequence is anticipated to result in a frameshift mutation, potentially introducing a premature stop codon into the new frame (Fig. 1C), thus producing truncated gene products (p.Val63GlyfsTer19). This particular genetic variant is absent in major population databases, including 1000 Genomes, ExAC, and gnomAD, suggesting its rarity and potential uniqueness to this family. The American College of Medical Genetics and Genomics (ACMG) classification system categorizes this mutation as “likely pathogenic” based on criteria PVS1 (null variant in a gene where loss of function is a known mechanism of disease) and PM2 (absent from controls in population databases), supporting the deleterious nature of this mutation.^[14]^ Additionally, another significant mutation, c.235delC, which is known to cause similar hearing loss phenotypes, was identified and inherited from the patient’s father.

At the age of 10 months, the patient underwent cochlear implantation. Although detailed examination results are not available for this study, her status was observed to remain stable throughout the follow-up period until she reached 5 years of age. However, maintaining patient follow-up became challenging thereafter.

## 3. Discussion

Mutations within the *GJB*2 gene can cause 2 types of deafness: autosomal recessive deafness DFNB1A and autosomal dominant deafness 3A (OMIM #601544). *GJB2*, initially associated with DFNB1A via mutations, remains a significant determinant in hereditary deafness across numerous populations.^[5,15–18]^ More than 400 unique variants of the *GJB2* gene have been identified, including missense, nonsense, frameshift, small in/del, and gross deletion mutations, with most resulting in DFNB1A.^[19,20]^ Recent studies further underscore the global prevalence and diverse impact of *GJB2* variants on hearing phenotypes, highlighting the gene’s allelic and genotypic variations that may influence the severity and type of hearing loss experienced by different ethnic groups.^[21]^ Variants that lead to a loss of function, such as c.35delG, c.235delC, and c.299_300delAT, are commonly associated with DFNB1A. In this study, we present a novel *GJB2* frameshift variant, c.188delT, resulting in a truncated protein (p.Val63GlyfsTer19), identified in compound heterozygosity with c.235delC in a deaf child from a non-consanguineous Chinese family. The truncated variant potentially induces harmful consequences, and the clinical manifestation of the patient is consistent with DFNB1A. The findings suggest a potential correlation between the c.188delT and c.235delC variants and the pathogenesis of DFNB1A.

Despite advancements in genetic screening and diagnosis, many patients with hereditary deafness remain undiagnosed, highlighting the complexity of the genetic factors underlying this condition. In our case, initial screening for common deafness genes revealed only 1 variant in the *GJB2* gene. However, further analysis using Sanger sequencing uncovered a novel truncating variant, c.188delT, in compound heterozygosity with c.235delC. Additionally, a variant c.2283A > G (p.Thr761=) was identified in the *SLC26A4* gene, but subsequent sequencing did not reveal any other mutations. Therefore, the inheritance pattern associated with *SLC26A4*-related diseases does not align with the clinical presentation observed in this case. The complex screening process and subsequent verification significantly delayed the diagnostic timeline, underscoring the necessity of comprehensive genetic testing to uncover the complete range of genetic mutations contributing to deafness. With the decreasing cost of high-throughput sequencing, Next-Generation Sequencing technology has the potential to replace the current traditional high-risk point screening technique based on the PCR hybridization method. It can expand the screening scope and significantly enhance the detection rate of deafness genes.^[22,23]^

The treatment of congenital deafness typically requires a multidisciplinary approach, which includes early intervention strategies like hearing aids, or cochlear implantation.^[24]^ This procedure has transformed the management of severe to profound sensorineural hearing loss, offering individuals with deafness the opportunity for sound perception and the development of spoken language skills. In the case presented, the patient received a cochlear implant at 10 months of age, leading to a consistent improvement in outcomes during the follow-up period until she reached 5 years of age. However, the challenges in maintaining patient follow-up beyond this point underscore the need for continued support and monitoring in the management of pediatric cochlear implant recipients. Additionally, research on Cx26-null mice suggests that genetic interventions, like inhibiting PARP-1, may mitigate hearing loss caused by *GJB2* mutations.^[25]^ This points to the potential integration of genetic therapy in treating congenital deafness, alongside conventional methods. Long-term surveillance is crucial to address device malfunctions, surgical complications, and evolving rehabilitation needs. Although *GJB2* gene therapy is still in development, advancements in genetic therapy hold promise for effective treatment of congenital deafness.^[26]^

In conclusion, our study has revealed a new variant, c.188delT, within the *GJB2* gene in a Chinese family with a newborn suffering from deafness. The discovery of novel variants like this broadens our understanding of the genetic factors underlying deafness and could guide future therapeutic strategies. However, the generalizability of our findings is limited by the study’s focus on a single case, which may not represent the full spectrum of genetic mutations associated with congenital hearing loss, and further clinical validation is required to confirm the pathogenicity of the mutation. Additionally, the follow-up duration was insufficient to assess long-term outcomes, highlighting the need for extended monitoring and research. Despite these limitations, our study emphasizes the importance of genetic counseling, prenatal and postnatal screening, and effective interventions for hearing loss. Sustained long-term monitoring and comprehensive care remain crucial in managing pediatric cochlear implant recipients, aiming to achieve the best possible outcomes and quality of life.

## Acknowledgments

We are grateful to the patient and her families in our research. We express our gratitude to all the pediatricians who helped with this study.

## Author contributions

**Conceptualization:** Yilun Tao.

**Data curation:** Yilun Tao, Zhipeng Hu.

**Formal analysis:** Yilun Tao, Zhipeng Hu, Dong Han, Haiwei Wang.

**Funding acquisition:** Yilun Tao.

**Investigation:** Lihong Wang, Haiwei Wang.

**Methodology:** Yilun Tao.

**Project administration:** Wenxia Song, Lihong Wang.

**Resources:** Dong Han.

**Supervision:** Xiaoze Li.

**Validation:** Wenxia Song.

**Writing – original draft:** Yilun Tao.

**Writing – review & editing:** Yilun Tao, Dong Han, Wenxia Song.
